# Hospital Clinics at the New York Hospital

**Published:** 1883-10

**Authors:** W. H. Draper

**Affiliations:** New York City


### ARTICLE VI.

HOSPITAL CLINICS, Given at the New York Hospital, by Prof. W. H. DRAPER, of New York City. Reported and arranged by W. F. Becker, M.D., Wauwatosa, Wis.

### No. 1. A CASE OF PSORA.

The patient is a male, æt. 17 years; native of Germany; occupation, sailor. He has always enjoyed excellent health. Two years ago, from sudden effort, sustained an inguinal hernia. No. history of gonorrhœa or syphilis. Temperate habits. Bowels have been regular, digestion and appetite good. Does not remember ever having eruptions when a child. Two weeks ago there appeared a vesicular and papular eruption between the fingers of left hand, with much irritation. At the same time there appeared a slight lump under left arm. Subsequently the right hand became the seat of a similar eruption, followed by the same condition at the inner side of thighs and elbows. The patient cannot account for the cause of the disorder.

*Examination.*—At the present the eruption does not exhibit any distinct individual character of skin lesion, for erythematous spots, vesicles, pustules, blebs, papules, superficial ulcers and scales are all present. Vesicles between the fingers are well marked, exist around wrist and arms; several are well marked on genitals, none on face or neck; scratch marks disguise the character of others.

*Remarks.*—The disease from which this man suffers is scabies, or psora, or itch, as it is commonly called; it belongs to the group of parasitic skin diseases. It depends upon the existence of a parasite called sarcoptus scabiei, or, more commonly, acarus scabiei, which produces a vesicular eruption. Later the eruption changes, assuming a polymorphous or multiform character, consequent upon irritation caused by its presence. In this patient the polymorphous form developed very rapidly, it being but two weeks since the disease began.

The history of this disease exhibits many curious facts, interesting from a scientific aspect. The acarus is an insect readily seen with the naked eye, which thrives on human integument, choosing for its invasion such parts where the skin is soft and tender, as on the insides of fingers and toes, in the flexures of joints, as about the ankle, wrist or axilla, on inner side of thighs, and genital organs. These regions are the seat of election of the disease. Sometimes in certain artisans the insect selects other parts of the integument, which are subject to pressure and warmth. Thus, in tailors or shoemakers, who sit much, or in men who ride much, the disease not infrequently develops in the skin of the buttocks. Usually, however, it begins on the hands, and only reaches other parts by their contact to those regions, generally next invading the genital organs, and later reaching other parts.

The insect does not thrive upon the surface of the integument, as do the cimex lectularius (bed bug), or pediculi, but bores its way into the epidermis, and makes its nest between the horny and mucous layers of the same, at a varying distance from the point where it penetrated. The canal thus formed is called the cuniculus. It can be detected only by close inspection, revealing itself by a grayish or whitish streak of a varying length, sometimes one-fourth of an inch or more. Here the female lays her eggs, and in the course of about ten days the eggs are matured, and reach the surface by the exfoliation of the epidermis. The young are there discharged, and meeting the male become impregnated, and in their turn burrow a new cuniculus, to repeat the process of their parent. The male acarus also burrows, but not so deeply as the female, only passing under the scales and crusts.

*Symptoms.*—The first symptom is that which is subjective and excites the patient's attention. It consists, as in this case, of a severe itching between the fingers. This irritation in some people of nervous temperament, is not confined to the part invaded by the parasite, but is universal—a general irritation existing, causing patients to scratch themselves everywhere; and the polymorphus form of lesion seen at the seat of the disorder is not caused by the ravages of the parasite, but subsequent and consequent to the scratching induced by the irritation of its presence.

The next symptom after the itching is the appearance of an eruption of a peculiar kind. This is, in its earliest stage, a vesicle with the acarian burrow or cuniculus running from it. This vesicle is after a time destroyed by scratching, and other lesions appear, following inflammation thus caused. The diagnosis is made from the polymorphous nature of the eruption, its location and the cuniculus, if seen. The crucial test, however, is the discovery of the acarus. This is done by inserting a needle through the cuniculus into the vesicle and turning out the insect. With the naked eye this has the appearance of a small white roundish body—under the microscope it develops more distinctive proportions. The discovery of the insect is often impossible, because of the destruction of its characteristic seat by scratching. It is claimed by many that without the discovery of the characteristic vesicle and the burrow leading from it, one is not justified in pronouncing the disease as scabies. This is too forcible an assertion, for sometimes the disease does not show this kind of vesicle; besides, it may be destroyed in the manner mentioned, and its distinctive character thus lost.

Scabies is contagious, as all parasitic skin diseases are, and it may be transmitted by contact with person or from wearing apparel. Its contagiousness to a certain extent depends on individual susceptibility. All persons are not equally susceptible to vegetable or animal parasites. It is necessary, besides the contagious element which they contain, that they have the proper host for their reception. The acari select their host—they do not find all congenial. Among the large number of persons surrounding the bed, many might shake the hand of the patient without being affected, while others could not do so with impunity.

*Treatment.*—This is very satisfactory when the diagnosis has been correctly made, but it must be carried out properly. The rational method is to remove the cause. For this a large number of drugs have been employed—carbolic acid, creasote, mercury, salts, and others; but of all parasiticides, sulphur seems to be the classical one for the disease. It is used in the form of baths, fumigation, or inunction. The last is the more frequent manner, and its details can be well set forth, perhaps, by describing the process adopted in Paris in the larger hospitals, where a large number of such cases are treated, particularly in the hospital of "St. Louis," which is devoted exclusively to treatment of skin diseases. Here there are several apartments. In the first the patients are undressed, and stand around a bin of soft soap. With this each patient rubs himself and his neighbor, the object being to soften the epidermis; then another apartment is entered, where they lie in hot baths for a period of from twenty minutes to half an hour. This is to still further favor the process of softening the integument. The third apartment contains in its center a tub, filled with sulphur ointment, in the proportion of about three parts of sulphur, one part carbonate of potash and eight parts of lard. Here the process of inunction is gone through with in very much the same manner as that of soaping; then the patients dress in their own clothing, which thus becomes disinfected. The entire process is repeated from three to five times, and the most obstinate cases are usually cured.

The same plan can be carried out in private practice. Care must be taken to make the application all over the body, as the acarus is migratory. The scalp, face and neck, where her incursions are scarcely ever found, are the only parts which may safely be excluded. The primary softening of the skin by the soaping and the bath is very important; without it the ointment is not efficient, for it opens the vesicles and the burrows and exposes their contents to the action of the sulphur. When the object of the treatment, viz., the destruction of the cause, is carefully adhered to, it generally gives much satisfaction.

